# Altered GDF15 and FGF21 Levels in Response to Strenuous Exercise: A Study in Marathon Runners

**DOI:** 10.3389/fphys.2020.550102

**Published:** 2020-11-19

**Authors:** Laura Campderrós, David Sánchez-Infantes, Joan Villarroya, Lexa Nescolarde, Antoni Bayès-Genis, Rubén Cereijo, Emma Roca, Francesc Villarroya

**Affiliations:** ^1^Departament de Bioquimica i Biomedicina Molecular, University of Barcelona, Barcelona, Spain; ^2^CIBER Fisiopatologia de la Obesidad y Nutrición, Madrid, Spain; ^3^Institut de Recerca Germans Trias i Pujol, Barcelona, Spain; ^4^Research Centre for Biomedical Engineering, Universitat Politècnica de Catalunya, Barcelona, Spain; ^5^Department of Electronic Engineering, Universitat Politècnica de Catalunya, Barcelona, Spain; ^6^Hospital Universitari Germans Trias i Pujol, Barcelona, Spain; ^7^Department of Medicine, Universitat Autònoma de Barcelona, Barcelona, Spain; ^8^CIBER de Enfermedades Cardiovasculares, Madrid, Spain

**Keywords:** strenuous exercise, GDF15, FGF21, marathon athletes, biomarker

## Abstract

**Background:**

Recreational marathon runners face strong physiological challenges. Assessment of potential biomarkers for the biological responses of runners will help to discriminate individual race responsiveness and their physiological consequences. This study sought to analyze the changes in the plasma levels of GDF15 and FGF21, novel endocrine factors related to metabolic stress, in runners following the strenuous exercise of a marathon race.

**Methods:**

Blood samples were obtained from eighteen male runners (mean ±SD, age: 41.7 ±5.0 years, BMI: 23.6 ± 1.8) 48 h before, immediately after, and 48 h after a marathon race, and from age-matched sedentary individuals. The level of GDF15, FGF21, and 38 additional biochemical and hematological parameters were determined.

**Results:**

The basal levels of GDF15 and FGF21 did not differ between runners before the race and sedentary individuals. Significant increases in the mean levels of GDF15 (4.2-fold) and FGF21 (20-fold) were found in runners immediately after the race. The magnitudes of these increases differed markedly among individuals and did not correlate with each other. The GDF15 and FGF21 levels had returned to the basal level 48 h post-race. The post-race value of GDF15 (but not FGF21) correlated positively with increased total white cell count (*r* = 0.50, *P* = 0.01) and neutrophilia (*r* = 0.10, *P* = 0.01).

**Conclusion:**

GDF15 and FGF21 are transiently increased in runners following a marathon race. The induction of GDF15 levels is associated with alterations in circulating immune cells levels.

## Introduction

Recreational running is a widespread activity and the marathon (a 42.2-km running race named for the Greek legend) is growing in popularity, with modern-day marathons drawing thousands of contestants. Recreational marathon runners face strong physiological challenges and multiple studies have reported alterations in physiological parameters and associated blood parameters during and after a marathon race (Kratz et al., 2002; Reid et al., 2004; Kobayashi et al., 2005; Bird et al., 2014). The commonly reported findings include increased levels of biomarkers for exertional myolysis as well as increased neutrophilia and monocytosis (Kratz et al., 2002; Bird et al., 2014). Additional biomarkers are needed to help discriminate differential individual race responsiveness.

Growth/differentiation factor-15 (GDF15), which is also called macrophage inhibitor (MIC-1) and non-steroidal anti-Inflammatory drug-activated gene-1 (NAG-1), is a member of the TGFβ superfamily. Although the functional role of GDF15 is not fully known, elevated GDF15 levels in blood have been reported as a biomarker of several pathological conditions, such as cancer, inflammatory disorders, cardiovascular disease and type 2 diabetes, and as a biomarker of mortality of any kind (Adela and Banerjee, 2015; Hagström et al., 2017; Tsai et al., 2018). The lack of an identified GDF15 receptor previously hampered efforts to assess the physiological role of GDF15 but recent work identified a receptor for GDF15 (GFRAL) in brain, and showed that it accounts for the anorexigenic actions of GDF15 (Emmerson et al., 2017; Hsu et al., 2017; Mullican et al., 2017; Yang et al., 2017). GDF15 has been reported to exert anti-inflammatory actions of GDF15 in peripheral cell systems, such as eosinophils and macrophages (Bootcov et al., 1997; Artz et al., 2016), via yet-unidentified receptor systems.

Elevated GDF15 levels have been reported as a biomarker of neuromuscular diseases of genetic origin caused by alterations in the mitochondrial genome (Fujita et al., 2015; Yatsuga et al., 2015). Indeed, experimentally induced alterations in muscle cell mitochondrial bioenergetics reportedly trigger enhancements in GDF15 gene expression and muscle cell release (Montero et al., 2016). In this sense, the secretion of GDF15 in muscle parallels that of fibroblast growth factor-21 (FGF21), which was recently proposed to be a biomarker of diseases caused by mitochondrial DNA mutations (Suomalainen et al., 2011; Ribas et al., 2014). In children affected by distinct mitochondrial diseases, the levels of GDF15 and FGF21 levels are increased and highly correlated with one another (Montero et al., 2016).

Numerous studies have reported that FGF21 levels increase in response to experimental acute exercise (Cuevas-Ramos et al., 2012; Kim et al., 2013; Hansen et al., 2015, 2016; Slusher et al., 2015; Tanimura et al., 2016; Morville et al., 2018; Sargeant et al., 2018). GDF15 has also been reported to exhibit transient elevations following an experimental single bout of exercise (Kleinert et al., 2018), a cycling race (Conte et al., 2020) and relatively time-limited sports activities such as a soccer match (Sanchis-Gomar et al., 2013) or a training session of rugby players (Galliera et al., 2014). Increased levels of GDF15 in athletes immediately after a 247-km race (the so-called Spartathlon) has also been reported (Tchou et al., 2009). However, the literature lacks a thorough characterization of changes in GDF15 and their comparison with FGF21 following a marathon race, which is the most commonly practiced recreational race involving strenuous exercise.

Given the potential roles of FGF21 and GDF15 in the physiological response to exercise, we wanted to explore whether these factors could contribute to the assessment of the physiological response to a marathon race. Here, we determined the levels of GDF15 and FGF21 in marathon runners before race (basal levels), immediately after the race (acute response to strenuous exercise) and 2 days after the race (short time recovery). We also sought to correlate these data with standard parameters previously reported to be altered in athletes after a marathon race (Kratz et al., 2002; Reid et al., 2004; Kobayashi et al., 2005; Bird et al., 2014).

## Methods

The study was designed to analyze the changes of GDF15 and FGF21 levels in recreational athletes before and after a marathon race held at Barcelona on March 17, 2017.

Volunteers were recruited as part of the “SUMMIT project” (“Health in Ultra-Marathon and their Limits”), which sought to evaluate the behavior of certain clinical parameters among runners competing in different races. The SUMMIT project was approved by an institutional review board (IIBSP-SUMMIT-2016-2) and all participants provided written informed consent to the current study. The study sample included 18 recreational male athletes (mean ± SD age: 41.7 ± 5.0 years, BMI: 23.6 ± 1.8). Sample size was dependent on availability of volunteers and was not based on power calculations; however, sample size was in the range of that resulting from power calculations in studies of individuals at rest and after a single bout of exercise (Yatsuga et al., 2015; Poulsen et al., 2020). The median (interquartile range, IQR) years of training was 7 (5–11) years and the median (IQR) of weekly training hours was 6 (5–8) h/week. The median (IQR) race time (h:min:s) was 3:32:44 (3:18:50–3:51:46).

The participants were provided with guidelines to maintain adequate levels of hydration during the race. The first liquid intake was programmed at 60 min of the race; the dosage consisted of 400 ml for lighter/slower runners and 800 ml for heavier/faster runners, and runners were asked to drink 100–150 ml every 15–20 min. Commercialized beverages were provided to participants; the drinks averaged 480 mg/L for Na^+^, 85 mg/L for K^+^, and 45 mg/L for Mg2^+^.

Three 10-mL blood samples were obtained from the antecubital vein in EDTA vacutainers at 48 h before the marathon (baseline), at completion (within the 10 min of an individual completing the race and before they drank any fluid or emptied the bladder) and 48 h after the race. Blood samples were centrifuged at 3,000 rpm at 4°C for 10 min in a bench-top centrifuge. Serum samples were aliquoted and stored on dry ice, and all samples were frozen at −80°C.

Biochemical and hematological parameters were determined in blood at the Clinical Biochemistry and Hematology facilities at Hospital Germans Trias i Pujol (Badalona, Spain). Blood glucose lipids, urea, total protein, ions, alanine aminotransferase (ALT), aspartate aminotransferase (AST), lactate dehydrogenase (LDH), and gamma-glutamyl transpeptidase (GGT) were analyzed by routine clinical chemistry and using an AU-5800 Chemistry Analyzer (Beckman Coulter Inc., Brea, CA, United States). Complete blood counts were obtained using a Unicel DxH800 automated hematology analyzer (Beckman Coulter, Miami, FL, United States). Troponin T was measured from serum, using a Highly Sensitive Troponin-T assay on a Cobas e601 platform (Roche Diagnostics, Barcelona, Spain). The inter-assays coefficients of variation (CV) in the analytical assays are shown in Supplementary Table 1. FGF21 levels (intra-assay CV 2.0%, inter-assay CV 3.3%) and GDF15 levels (intra-assay CV 2.6%, inter-assay CV 5.3%) were measured using human-specific ELISA kits (R&D Systems, United Kingdom, and Biovendor, Czechia, respectively). For comparison purposes, an age-matched and BMI-matched group of healthy male volunteers, with a sedentary behavioral profile (no recreational or relevant occupational-related exercise activity), was recruited among personnel of the University of Barcelona (*N* = 19, mean ± SD age: 41.4 ± 3.0 years) and studied.

The normality of distribution of the variables was checked by the Shapiro–Wilk test and the homogeneity of variances was assessed by Levene’s test. Repeated-measures ANOVA test was used to determine the effect of the marathon on variables measured at 24 h pre-race, immediately post-race and 48 h post-race using the multiple-comparison Bonferroni tests. Pearson correlation coefficient was applied according to the normal distribution of variables. The level of statistical significance was set at *P* < 0.05. IBM^®^ SPSS^®^ version 24.0 (Armonk, NY, United States) was used for data analysis.

## Results

Immediately after the race, the participating runners showed an increase in their total white blood cell (WBC) count; this was associated with increased numbers of neutrophils, monocytes, and basophils but reduced numbers of lymphocytes and eosinophils (Table 1). These changes were totally reversed at 48 h after the race, when the values for these parameters were not statistically different from the basal values. Significant increases were also observed in the levels of urea, creatinine, calcium, total protein, sodium, potassium, and bilirubin immediately after the race; all of these parameters also returned to basal levels with the exception of potassium, which remained high at 48 h post-race, and total protein which was decreased below the basal level at 48 h post-race. The magnesium and phosphorus levels were transiently reduced after the race and normalized totally (magnesium) or partially (phosphorus) 48 h later. The levels of LDH and troponin were significantly induced immediately after the race; at 48 h after the race, troponin had returned to the basal level whereas LDH remained significantly elevated. The creatine kinase level was significantly higher immediately after the race compared to basal level and was further increased at 48 h post-race. Finally, the levels of ALT and C-reactive protein levels were increased relative to their basal levels only at 48 h post-race.

**TABLE 1:** Circulating parameters measured before (pre-marathon), immediately after (post-marathon) and 48 h after the marathon race.

	Pre-marathon	Post-marathon	48 h Post-marathon
Age (years)	41.71 ± 1.04		
Weight (kg)	76.15 ± 1.35		
White blood cell count (±10^9^/L)	6.28 ± 0.30	13.55 ± 0.65***	**5.70 ± 0.30^###^**
Red blood cell count (±10^6^/μL)	5.06 ± 0.10	5.04 ± 0.09	4.89 ± 0.09
Mean corpuscular hemoglobin concentration (μ/dL)	33.25 ± 0.13	33.33 ± 0.11	32.77 ± 0.14*^##^
Hemoglobin (g/dL)	14.64 ± 0.21	14.63 ± 0.18	14.02 ± 0.17*
Hematocrit (%)	44.06 ± 0.62	43.86 ± 0.54	42.80 ± 0.50
Mean corpuscular volume (fL)	87.44 ± 0.91	87.27 ± 0.86	87.83 ± 0.86
Mean corpuscular hemoglobin (pg)	29.09 ± 0.37	29.09 ± 0.36	28.81 ± 0.36
Platelets (x10^3^/μL)	188.30 ± 7.34	218.30 ± 10.23*	**184.30 ± 7.93^#^**
Neutrophils (x10^9^/L)	3.47 ± 0.20	11.41 ± 0.60***	**3.06 ± 0.17^###^**
Lymphocytes (x10^9^/L)	2.00 ± 0.08	1.16 ± 0.07***	**1.84 ± 0.11^###^**
Monocytes (x10^9^/L)	0.46 ± 0.02	0.78 ± 0.06***	**0.43 ± 0.03^###^**
Eosinophils (x10^9^/L)	0.16 ± 0.02	0.02 ± 0.007***	**0.14 ± 0.02^###^**
Basophils (x10^9^/L)	0.02 ± 0.01	0.06 ± 0.01*	**0.01 ± 0.006^###^**
Glucose (mg/dL)	85.96 ± 2.26	85.96 ± 4.11	81.62 ± 2.26
Urea (mg/dL)	35.96 ± 1.80	42.63 ± 1.62*	**35.26 ± 1.47^##^**
Creatinine (mg/dL)	0.96 ± 0.02	1.25 ± 0.04***	**0.93 ± 0.02^###^**
Calcium (mg/dL)	9.42 ± 0.06	9.92 ± 0.09***	**9.29 ± 0.06^###^**
Magnesium (mmol/L)	2.02 ± 0.03	1.77 ± 0.03***	**2.05 ± 0.03^###^**
Phosphorus (mg/dL)	3.58 ± 0.11	2.86 ± 0.15***	3.16 ± 0.09*
Total protein (g/L)	72.72 ± 0.63	76.59 ± 0.76***	69.54 ± 0.54**^###^
Sodium (mmol/L)	139.20f ± 0.20	141.90 ± 0.51***	**139.10 ± 0.26^###^**
Potassium (mmol/L)	4.24 ± 0.05	4.45 ± 0.06**	4.41 ± 0.05*
Total bilirubin (mg/dL)	0.69 ± 0.04	0.88 ± 0.04**	**0.72 ± 0.03 ^#^**
Alkaline phosphatase (U/L)	74.12 ± 3.07	78.81 ± 3.36	69.0 ± 3.52
Gamma-glutamyl transpeptidase (U/L)	20.12 ± 1.65	20.69 ± 1.43	18.62 ± 1.26
Alanine aminotransferase (U/L)	22.59 ± 1.70	24.44 ± 1.57	33.73 ± 2.71***^##^
Lactate dehydrogenase (U/L)	186.6 ± 5.03	324.7 ± 14.50***	228.7 ± 9.00*^###^
Creatine kinase (U/L)	170.3 ± 15.58	397.0 ± 28.99*	802.6 ± 107***^###^
Troponin (ng/L)	3.92 ± 0.67	31.88 ± 5.03***	**5.87 ± 0.88^###^**
C-reactive protein (mg/dL)	0.89 ± 0.15	0.64 ± 0.09	6.81 ± 0.78***^###^
Triglycerides (mg/dL)	105.3 ± 8.85	100.4 ± 5.67	82.15 ± 8.81
Cholesterol (mg/dL)	186.3 ± 7.43	185.0 ± 6.60	172.9 ± 6.13
GDF15 (pg/mL)	372.8 ± 21.20	1,164 ± 124.7***	**347.6 ± 15.82^###^**
FGF21 (pg/mL)	20.24 ± 7.33	770.3 ± 130.2***	**39.52 ± 11.77^###^**

*P < 0.05, **P < 0.01, and ***P < 0.001 relative to the measurements before the race and ^#^*P* < 0.05, ^##^*P* < 0.01, and ^###^*P* < 0.001 relative to the measurements immediately after the race. Bold lettering is shown when *P* < 0.05.

**FIGURE 1:**
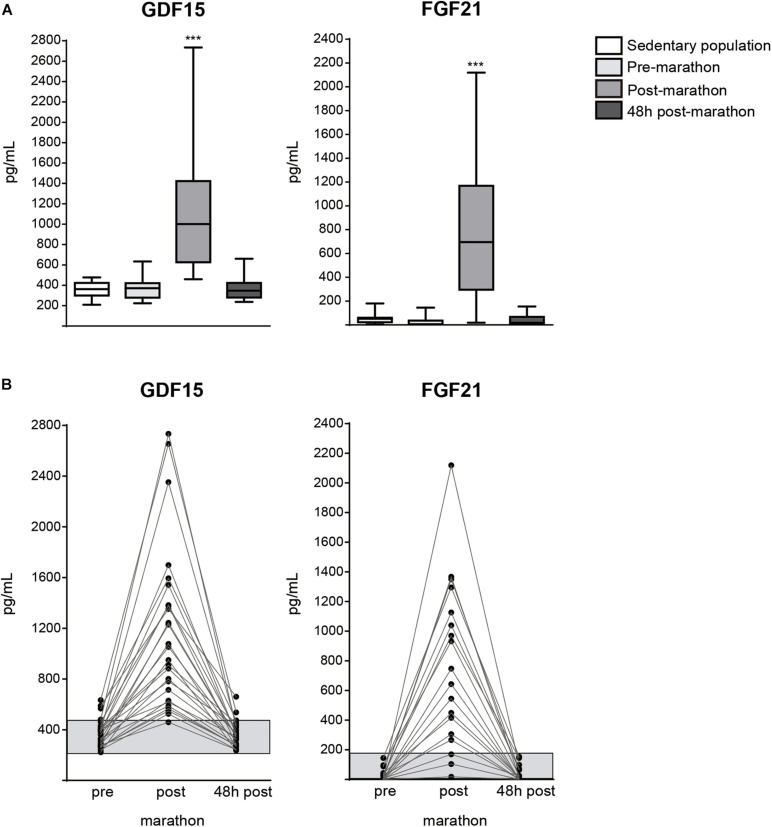
Levels of GDF15 and FGF21 in marathon runners 48 h before the race (pre-marathon), immediately after the race (post-marathon) and 48 h after the race, and comparison with levels in a sedentary control population. **(A)** Box and whisker plot representation of GDF15 and FGF21 levels, ***P < 0.001 relative to pre-marathon values. **(B)** Individual changes in the levels of GDF15 and FGF21 in the three time points analyzed. Gray bar corresponds to means + SEM values in the sedentary control population.

The basal levels of plasma GDF15 and FGF21 did not significantly differ between pre-race athletes and sedentary individuals (Figure 1A), indicating that chronic training does not influence the basal levels of these two bioactive factors. Immediately after the race, the levels of both GDF15 and FGF21 levels were dramatically increased in athletes; there was a 4.2-fold increase in the mean GDF15 level and a ∼20-fold increase in the mean FGF21 level (Figure 1A), and the extents of these increases differed markedly among the studied individuals (Figure 1B). The plasma levels of GDF15 and FGF21 had returned to near-basal levels at 48 h post-race. There was no significant correlation in the basal levels of GDF15 and FGF21 or the extent of their increases immediately after the race (Table 2). There was also no significant correlation between the increase of GDF15 or FGF21 and the time spent in the race or any other race-related variable. Among the 32 distinct physiological and blood biochemical and hematological parameters tested herein, the basal (pre-race) level of GDF15 was positively correlated with that of ALT (Supplementary Table 2) and the GDF15 level immediately after the race was positively correlated with the WBC count, neutrophil count, urea level, and ALT level (Table 2). The basal levels of FGF21 correlated positively with the WBC count, platelet count, monocyte count, total protein level, and C-reactive protein level (Supplementary Table 2), and correlated negatively with the urea level. The peak levels of FGF21 observed just after the race correlated positively with glycemia and the total protein levels, with marginal statistically significance (Table 2).

**TABLE 2:** Linear relationship of the post-marathon GDF15 levels and FGF21 levels with the post-marathon levels of circulating parameters.

	GDF15		FGF21	
	*r*	*P*	*r*	*P*
GDF15	–	–	0.394	0.106
FGF21	0.394	0.106	–	–
White blood cell count (×10^9^/L)	**0.455**	0.022*	0.930	0.237
Red blood cell count (×10^6^/μL)	−0.067	0.751	−0.237	0.343
Mean corpuscular hemoglobin concentration (g/dL)	−0.129	0.539	0.061	0.811
Hemoglobin (g/dL)	−0.054	0.799	−0.202	0.422
Hematocrit (%)	−0.015	0.942	−0.217	0.387
Mean corpuscular volume (fL)	0.113	0.591	0.149	0.555
Mean corpuscular hemoglobin (pg)	0.058	0.782	0.134	0.596
Platelets (×10^3^/μL)	0.084	0.689	0.374	0.126
Neutrophils (×10^9^/L)	**0.506**	0.010**	0.314	0.205
Lymphocytes (×10^9^/L)	−0.225	0.279	−0.059	0.816
Monocytes (×10^9^/L)	0.326	0.112	0.319	0.198
Eosinophils (×10^9^/L)	−0.368	0.071	−0.152	0.547
Basophils (×10^9^/L)	0.241	0.247	0.050	0.843
Glucose (mg/dL)	−0.136	0.518	**−0.470**	0.049*
Urea (mg/dL)	**0.661**	0.0003***	0.004	0.989
Creatinine (mg/dL)	0.349	0.087	0.103	0.684
Calcium (mg/dL)	0.122	0.560	0.155	0.539
Magnesium (mmol/L)	0.062	0.770	−0.038	0.881
Phosphorus (mg/dL)	−0.129	0.541	−0.461	0.055
Total protein (g/L)	0.140	0.504	**0.479**	0.044*
Sodium (mmol/L)	−0.166	0.429	−0.069	0.786
Potassium (mmol/L)	0.373	0.066	0.212	0.398
Total bilirubin (mg/dL)	0.214	0.305	−0.054	0.832
Alkaline phosphatase (U/L)	0.230	0.268	−0.238	0.341
Gamma-glutamyl transpeptidase (U/L)	−0.064	0.761	0.325	0.189
Alanine aminotransferase (U/L)	**0.498**	0.011*	0.240	0.338
Lactate dehydrogenase (U/L)	0.157	0.452	0.156	0.536
Creatine kinase (U/L)	−0.227	0.275	−0.382	0.118
Troponin (ng/L)	0.142	0.498	−0.236	0.345
C-reactive protein (mg/dL)	0.011	0.958	0.176	0.485
Triglycerides (mg/dL)	0.162	0.439	0.304	0.220
Cholesterol (mg/dL)	−0.219	0.292	−0.255	0.308

Statistical significance is from Pearson correlation test. *P < 0.05, **P < 0.01, and ***P < 0.001. Bold lettering is shown when *P* < 0.05.

## Discussion

Overall, the alterations in blood parameters observed in the current study are largely concordant with previous findings on the physiological response to marathon running. We found increases in the levels of total protein, urea, creatinine, bilirubin, creatine kinase, LDH, and troponin after the race; these findings paralleled those of a previous study in marathon runners (Bird et al., 2014) and were consistent with a scenario of exertional muscle injury and hemolysis. Some of these parameters returned to their basal values by 48 h post-race, whereas others (e.g., creatine kinase, LDH, and troponin) did not; again, this was consistent with previous reports (Kobayashi et al., 2005; Bird et al., 2014). We also confirmed that the increase in total WBC count was mainly due to increased numbers of neutrophils and monocytes (Kratz et al., 2002; Reid et al., 2004), which is commonly attributed to an inflammatory reaction to exertion-related tissue injury.

Regarding our novel results, we herein report that the plasma levels of GDF15 and FGF21 were both increased immediately after the marathon race and returned to normal levels within 48 h post-race. The extent of these transient bursts in GDF15 and FGF21 concentration was very variable among individuals, was not associated with any of the studied intrinsic exercise-associated traits (i.e., extent of prior training, duration of the race, etc.), and the degrees to which GDF15 and FGF21 increased were not correlated with each other in studied runners.

This is one of only a few studies to focus on how exercise affect the level of GDF15. Studies performed in soccer players after a match and rugby players after training reported a significant increase in GDF15 levels in blood (Sanchis-Gomar et al., 2013; Galliera et al., 2014), and a recent experimental study showed that there was a significant rise in GDF15 levels among volunteers who exercised at 67% of their VO2max for 1 h (Kleinert et al., 2018). In these reports, the increase in GDF15 was around 1.5- to 2-fold, which is much less than the more than fourfold increase found here after marathon race-associated strenuous exercise. Only a study in spartathlon runner found a rise in GDF15 levels in the fourfold range found here for marathon runners (Tchou et al., 2009) whereas around threefold increase in GDF51 levels were reported after a long distance cycling race (Conte et al., 2020). During the editorial processing of the current article, a study in Marathon runners in Poland has been reported (Kaleta-Duss et al., 2020) showing a rise in GDF15 levels immediately after the race and normalization thereafter, confirming our findings. In that study, focused to cardiovascular markers, a significant increase in the biomarkers of altered cardiac function BNP, NT-proANP, H-FABP, and Gal-3 immediately after the race was also found; however, they did not correlate with GDF15. Although the rise in GDF15 may be related to changes in cardiac hemodynamic volume and pressure overload immediately after the marathon, it has been proposed that GDF15 does not appear as reliable to track specifically cardiorespiratory fitness in acute exercise, possibly because of the contribution of other non-cardiac processes to the GDF15 rise in exercise (Kaleta-Duss et al., 2020). Nonetheless, some authors found GDF15 levels to be associated with impairment in exercise capacity in patients with the heart failure syndrome (Stahrenberg et al., 2010) whereas others do not consider GDF15 as a reliable biomarker of exercise capacity in heart failure patients (Fudim et al., 2020).

Concerning FGF21, the previous studies reported –twofold to fourfold increases in FGF21 after experimental bouts of acute exercise (1–3 h) (Kim et al., 2013; Hansen et al., 2015, 2016; Slusher et al., 2015; Morville et al., 2018; Sargeant et al., 2018), whereas we observed a much greater increase of around 20-fold in the level of FGF21 immediately after the runners completed the marathon race.

It is worth mentioning that, beyond exercise, the blood levels of GDF15 and FGF21 are also increased in pathological contexts, such as in patients affected by neuromuscular diseases caused by mitochondria DNA mutations (Suomalainen et al., 2011). Moreover, GDF15 and FGF21 were reported to be actively released by human muscle cells after experimental mitochondrial insults *in vitro* (Ribas et al., 2014; Montero et al., 2016). However, not all the above mentioned studies found correlations between the high levels of GDF15 and FGF21 in patients, and it has been proposed that GDF15 may have value as a biomarker of muscle damage, whereas FGF21 may be more strongly associated with mitochondrial-specific pathologies (Lehtonen et al., 2016).

The lack of correlation between the bursts of the two molecules reported here suggests that distinct physiological processes (and possibly tissues of origin) are involved in these increases. Given that the increase in FGF21 is associated with decreased glycemia and the liver is the main site of FGF21 release under physiological conditions, it is likely that the liver was the main source of the FGF21 increase seen immediately after the marathon race. This has been proposed for other exercise-induced changes in FGF21 levels and it is consistently with the behavior of other hepatokines (Weigert et al., 2019). For GDF15, a recent study indicated that the rise in GDF15 after a single bout of exercise is associated with increased GDF15 gene expression in skeletal muscle (Laurens et al., 2020). From this, we hypothesize that muscle may be the source of the high-level induction of GDF15 seen immediately after the marathon race, although correlation with ALT levels does not allow to rule out a potential role of hepatic stress contributing to high GDF15 levels. Further studies are clearly required in order to identify the tissue sources that lead to the rise in GDF15 and FGF21 levels in marathon runners. This may be relevant in order to identify critical sites of tissue stress due to the race indistinct individuals according to their rise in the GDF15 and/or FGF21 biomarkers.

Our study has several limitations. The sample is relatively small and limited to recreational runners, and further data on the characteristics of individuals beyond biochemical and hematological data (e.g., body composition, performance level, pacing, and internal load) could have strengthened the study. The correlative nature of some of our findings is also an obvious limitation. However, our data allow us to conclude that: (a) both GDF15 and FGF21 are dramatically induced immediately after the strenuous exercise associated with a marathon race; (b) the extents to which GDF15 and FGF21 are induced are highly variable among runners and do not correlate with each other; (c) both parameters recover to baseline within 2 days post-race; and (d) the rises in GDF15 and FGF21 immediately after the race are distinctly correlated with alterations in biochemical and hematological biomarkers of the physiological response to exercise. Further research is needed to ascertain whether the strong intra-individual differences in the extent of GDF15 and FGF21 induction among runners can predict underlying adaptations and/or the risk for long-term damaging responses to the strenuous exercise associated with a marathon race.

## Data Availability Statement

The raw data supporting the conclusions of this article will be made available by the authors, without undue reservation.

## Ethics Statement

The studies involving human participants were reviewed and approved by Comitè Ètic de l’Hospital Universitari Germans Trias i Pujol (ICOR-2017- 04, REF.CEI, PI-17-037). The patients/participants provided their written informed consent to participate in this study.

## Author Contributions

ER, AB-G, and FV designed the study. DS-I and RC obtained the samples. LC, JV, and LN performed the analytical procedures. ER and FV wrote the manuscript. All authors discussed the data.

## Conflict of Interest

The authors declare that the research was conducted in the absence of any commercial or financial relationships that could be construed as a potential conflict of interest.
