# Supplementary material for: Altered GDF15 and FGF21 Levels in Response to Strenuous Exercise: A Study in Marathon Runners
Source: Front Physiol. 2020 Nov 19;11:550102. doi: 10.3389/fphys.2020.550102 (PMC7711067; doi:10.3389/fphys.2020.550102)
Supplement: Supplementary file 1 [file Data_Sheet_1.docx]

**Supplementary table 1. Inter-assay coefficients of variation of biochemical parameters.**

| Glucose (mg/dL) | 2,2% |
| --- | --- |
| Urea (mg/dL) | 3,4% |
| Creatinine (mg/dL) | 4,8% |
| Calcium (mg/dL) | 1.4% |
| Magnesium (mmol/L) | 4,4% |
| Phosphorus (mg/dL) | 2,7% |
| Total protein (g/L) | 1,5% |
| Sodium (mmol/L) | 0,9% |
| Potassium (mmol/L) | 1,4% |
| Total bilirubin (mg/dL) | 2,6% |
| Alkaline phosphatase (U/L) | 2,3% |
| Gamma-glutamyl transpeptidase (U/L) | 2,1% |
| Alanine aminotransferase (U/L) | 2,3% |
| Lactate dehydrogenase (U/L) | 4,4% |
| Creatine kinase (U/L) | 3,6% |
| Troponin (ng/L) | 3,9% |
| C-reactive protein (mg/dL) | 2,3% |
| Triglycerides (mg/dL) | 1,8% |
| Cholesterol (mg/dL) | 2,2% |

**Supplementary table 2. Linear relationship of the pre-marathon GDF15 levels and FGF21 levels with the pre-Marathon levels of circulating parameters.**

|  | GDF15 | | FGF21 | | |
| --- | --- | --- | --- | --- | --- |
|  | r | p | | r | p |
| GDF15 | - | - | | -0.302 | 0.234 |
| FGF21 | -0.297 | 0.232 | | - | - |
| White blood cell count (x10^9^/L) | 0.0148 | 0.944 | | **0.518** | **0.028*** |
| Red blood cell count (x10^6^/uL) | 0.0719 | 0.733 | | 0.169 | 0.503 |
| Mean corpuscular hemoglobin concentration (g/dL) | -0.376 | 0.064 | | -0.052 | 0.838 |
| Hemoglobin (g/dL) | -0.119 | 0.572 | | -0.001 | 0.995 |
| Hematocrit (%) | -0.010 | 0.962 | | -0.005 | 0.984 |
| Mean corpuscular volume (fL) | -0.111 | 0.600 | | -0.307 | 0.216 |
| Mean corpuscular hemoglobin (pg) | -0.205 | 0.326 | | -0.259 | 0.299 |
| Platelets (x10^3^/µL) | 0.0189 | 0.930 | | **0.521** | **0.027*** |
| Neutrophils (x10^9^/L) | 0.072 | 0.734 | | 0.351 | 0.154 |
| Lymphocytes (x10^9^/L) | -0.090 | 0.669 | | 0.466 | 0.051 |
| Monocytes (x10^9^/L) | -0.160 | 0.445 | | **0.534** | **0.023*** |
| Eosinophils (x10^9^/L) | -0.003 | 0.989 | | 0.078 | 0.760 |
| Basophils (x10^9^/L) | 0.039 | 0.853 | | 0.452 | 0.060 |
| Glucose (mg/dL) | -0.276 | 0.181 | | -0.146 | 0.562 |
| Urea (mg/dL) | 0.186 | 0.373 | | **-0.520** | **0.027*** |
| Creatinine (mg/dL) | -0.227 | 0.275 | | -0.310 | 0.211 |
| Calcium (mg/dL) | 0.132 | 0.531 | | 0.189 | 0.454 |
| Magnesium (mmol/L) | 0.024 | 0.908 | | -0.008 | 0.976 |
| Phosphorus (mg/dL) | -0.043 | 0.839 | | -0.255 | 0.308 |
| Total protein (g/L) | 0.061 | 0.772 | | **0.612** | **0.007**** |
| Sodium (mmol/L) | 0.125 | 0.551 | | -0.168 | 0.506 |
| Potassium (mmol/L) | -0.020 | 0.926 | | 0.093 | 0.714 |
| Total bilirubin (mg/dL) | -0.234 | 0.259 | | 0.123 | 0.626 |
| Alkaline phosphatase (U/L) | 0.003 | 0.989 | | -0.152 | 0.548 |
| Gamma-glutamyl transpeptidase (U/L) | 0.073 | 0.730 | | -0.042 | 0.869 |
| Alanine aminotransferase (U/L) | **0.438** | **0.029*** | | -0.280 | 0.260 |
| Lactate dehydrogenase (U/L) | 0.211 | 0.311 | | -0.301 | 0.218 |
| Creatine kinase (U/L) | 0.242 | 0.244 | | -0.181 | 0.473 |
| Troponin (ng/L) | -0.044 | 0.834 | | 0.185 | 0.464 |
| C-reactive protein (mg/dL) | -0.039 | 0.852 | | **0.617** | **0.006**** |
| Triglycerides (mg/dL) | -0.241 | 0.247 | | 0.337 | 0.172 |
| Cholesterol (mg/dL) | -0.021 | 0.920 | | 0.075 | 0.768 |

Statistical significance is from Pearson correlation test. Bold lettering is shown when P < 0,05.

**Supplementary table 3. Linear relationship of the 48h post-marathon GDF15 levels and FGF21 levels with the 48h post-Marathon levels of circulating parameters.**

|  | GDF15 | | FGF21 | |
| --- | --- | --- | --- | --- |
|  | r | p | r | p |
| GDF15 | - | - | -0,012 | 0,963 |
| FGF21 | -0.012 | 0.963 | - | - |
| White blood cell count (x10^9^/L) | 0.183 | 0.381 | 0,133 | 0,598 |
| Red blood cell count (x10^6^/uL) | 0.147 | 0.482 | -0,127 | 0,615 |
| Mean corpuscular hemoglobin concentration (g/dL) | -0.110 | 0.600 | -0,112 | 0,659 |
| Hemoglobin (g/dL) | -0.060 | 0.774 | -0,123 | 0,628 |
| Hematocrit (%) | -0.008 | 0.970 | -0,093 | 0,712 |
| Mean corpuscular volume (fL) | -0.233 | 0.261 | 0,118 | 0,641 |
| Mean corpuscular hemoglobin (pg) | -0.224 | 0.282 | 0,047 | 0,854 |
| Platelets (x10^3^/µL) | 0.093 | 0.657 | 0,1314 | 0,6033 |
| Neutrophils (x10^9^/L) | 0.246 | 0.235 | 0,155 | 0,539 |
| Lymphocytes (x10^9^/L) | -0.041 | 0.846 | -0,009 | 0,971 |
| Monocytes (x10^9^/L) | -0.038 | 0.856 | 0,055 | 0,830 |
| Eosinophils (x10^9^/L) | 0.004 | 0.984 | 0,026 | 0,918 |
| Basophils (x10^9^/L) | 0.361 | 0.076 | 0,133 | 0,600 |
| Glucose (mg/dL) | 0.234 | 0.260 | -0,424 | 0,080 |
| Urea (mg/dL) | 0.019 | 0.929 | -0,364 | 0,138 |
| Creatinine (mg/dL) | -0.168 | 0.422 | **-0,490** | **0,039*** |
| Calcium (mg/dL) | -0.071 | 0.735 | -0,094 | 0,710 |
| Magnesium (mmol/L) | -0.064 | 0.761 | 0,264 | 0,290 |
| Phosphorus (mg/dL) | -0.375 | 0.065 | 0,118 | 0,641 |
| Total protein (g/L) | -0.306 | 0.136 | 0,377 | 0,123 |
| Sodium (mmol/L) | -0.072 | 0.734 | 0,081 | 0,749 |
| Potassium (mmol/L) | -0.076 | 0.720 | -0,148 | 0,558 |
| Total bilirubin (mg/dL) | -0.139 | 0.508 | -0,169 | 0,502 |
| Alkaline phosphatase (U/L) | 0.041 | 0.844 | -0,063 | 0,803 |
| Gamma-glutamyl transpeptidase (U/L) | -0.124 | 0.556 | **0,503** | **0,034*** |
| Alanine aminotransferase (U/L) | -0.217 | 0.298 | 0,343 | 0,164 |
| Lactate dehydrogenase (U/L) | -0.354 | 0.083 | 0,268 | 0,282 |
| Creatine kinase (U/L) | -0.312 | 0.129 | 0,332 | 0,179 |
| Troponin (ng/L)) | -0.016 | 0.939 | -0,203 | 0,420 |
| C-reactive protein (mg/dL) | 0,054 | 0,797 | 0,139 | 0,583 |
| Triglycerides (mg/dL) | -0,029 | 0,889 | 0,337 | 0,172 |
| Cholesterol (mg/dL) | -0,093 | 0,659 | 0,126 | 0,618 |

Statistical significance is from Pearson correlation test. Bold lettering is shown when P < 0,05.
